# Hba1c levels are increased in patients with gestational diabetes carrying the T/T genotype of the rs1990760 polymorphism in the IFIH1 gene

**DOI:** 10.1186/1758-5996-7-S1-A75

**Published:** 2015-11-11

**Authors:** Ana Paula Bouças Kochenborger, Ana Paula Kutscher Ripoll, Bianca Marmontel de Souza, Pamela Sachs Nique, Denise Alves Sortica, Natali Cardoso, Sandra Pinho Silveiro, Leticia Schwerz Weinert, Sergio Hofmeister de Almeida Martins Costa, Luís Henrique Canani, Daisy Crispim

**Affiliations:** 1 HCPA, Porto Alegre, Brazil

## Background

Gestational diabetes mellitus (GDM) is diabetes diagnosed in the second or third trimester of pregnancy that is not clearly overt diabetes. This condition is a common complication of pregnancy, being associated with both maternal and neonatal adverse outcomes. Several studies have indicated that viruses play an important role in the pathophysiology of diabetes. IFIH1/MDA5 gene encodes a cytoplasmic receptor that recognizes viral nucleic acids, playing a major role in the immune response against viruses. Accordingly, the rs1990760 (G/A) polymorphism in the IFIH1 gene has been associated with type 1 diabetes mellitus in different populations. Considering that the MDA5 receptor is expressed in human placenta, we therefore hypothesized that the rs1990760 G/A polymorphism could be also associated with GDM.

## Objectives

To investigate the association between the IFIH1 rs1990760 polymorphism and GDM and/or its clinical characteristics in a Southern Brazilian population. Moreover, we aimed to analyze IFIH1 expression in placentas from GDM patients and healthy pregnant women according to different rs1990760 genotypes.

## Materials and methods

We analyzed 129 patients with GDM (cases) and 144 pregnant women without GDM (controls). The polymorphism was genotyped by RT-PCR using TaqMan MGB probes (Life Technologies). IFIH1 expressions in placentas from 70 cases and 36 controls were evaluated using RT-qPCR.

## Results

Genotype and allele frequencies of the rs1990760 polymorphism did not differ between GDM patients and nondiabetic controls (P=0.702 and P=0.708, respectively), and adjustment for ethnicity did not change these Results. In GDM patients, fasting glucose levels, body mass index and age were not significantly different between rs1990760 genotypes. However, T/T genotype carriers had increased levels of glycated hemoglobin (A1c) as compared to G allele carriers (5.9 ± 0.4 vs. 5.4 ± 0.5, P=0.007). IFIH1 expression in placenta was similar among the three genotypes of the rs1990760 polymorphism (P>0.05). Interestingly, IFIH1 expression in placenta was decreased in patients with GDM as compared to controls (7.0 ± 4.2 vs. 9.7 ± 9.4, P=0.044). Accordingly, IFIH1 expression was inversely correlated to A1c levels (r=0.549, P=0.035).

## Conclusions

This study suggests an association between the T/T genotype with increased levels of A1c. Furthermore, IFIH1 gene expression seems to be associated with protection for GDM, and it was inversely correlated to A1c levels.

